# A Computerized Method for Measuring Computed Tomography Pulmonary Angiography Yield in the Emergency Department: Validation Study

**DOI:** 10.2196/medinform.9957

**Published:** 2018-10-25

**Authors:** Safiya Richardson, Philip Solomon, Alexander O'Connell, Sundas Khan, Jonathan Gong, Alex Makhnevich, Guang Qiu, Meng Zhang, Thomas McGinn

**Affiliations:** 1 Department of Medicine Donald and Barbara Zucker School of Medicine at Hofstra/Northwell Hempstead, NY United States

**Keywords:** health informatics, pulmonary embolism, electronic health record, quality improvement, clinical decision support systems

## Abstract

**Background:**

Use of computed tomography pulmonary angiography (CTPA) in the assessment of pulmonary embolism (PE) has markedly increased over the past two decades. While this technology has improved the accuracy of radiological testing for PE, CTPA also carries the risk of substantial iatrogenic harm. Each CTPA carries a 14% risk of contrast-induced nephropathy and a lifetime malignancy risk that can be as high as 2.76%. The appropriate use of CTPA can be estimated by monitoring the CTPA yield, the percentage of tests positive for PE. This is the first study to propose and validate a computerized method for measuring the CTPA yield in the emergency department (ED).

**Objective:**

The objective of our study was to assess the validity of a novel computerized method of calculating the CTPA yield in the ED.

**Methods:**

The electronic health record databases at two tertiary care academic hospitals were queried for CTPA orders completed in the ED over 1-month periods. These visits were linked with an inpatient admission with a discharge diagnosis of PE based on the International Classification of Diseases codes. The computerized the CTPA yield was calculated as the number of CTPA orders with an associated inpatient discharge diagnosis of PE divided by the total number of orders for completed CTPA. This computerized method was then validated by 2 independent reviewers performing a manual chart review, which included reading the free-text radiology reports for each CTPA.

**Results:**

A total of 349 CTPA orders were completed during the 1-month periods at the two institutions. Of them, acute PE was diagnosed on CTPA in 28 studies, with a CTPA yield of 7.7%. The computerized method correctly identified 27 of 28 scans positive for PE. The one discordant scan was tied to a patient who was discharged directly from the ED and, as a result, never received an inpatient discharge diagnosis.

**Conclusions:**

This is the first successful validation study of a computerized method for calculating the CTPA yield in the ED. This method for data extraction allows for an accurate determination of the CTPA yield and is more efficient than manual chart review. With this ability, health care systems can monitor the appropriate use of CTPA and the effect of interventions to reduce overuse and decrease preventable iatrogenic harm.

## Introduction

The ability of computed tomography (CT) to diagnose pulmonary embolism (PE) was demonstrated in 1980 [1]. The introduction of multidetector row CT pulmonary angiography (CTPA) revolutionized the diagnostic approach to PE in 1998 [2]. The availability and use of this new technology rapidly increased in the following years, and by 2001, CT overtook the ventilation/perfusion lung (V/Q) scan as the most common method for diagnosing PE [3].

In 2006, results from the landmark prospective investigation of pulmonary embolism diagnosis (PIOPED) II trial established CTPA as the first-choice diagnostic imaging modality, with a sensitivity of >90% for patients with high clinical suspicion of PE and a specificity of 96% [4,5]. Over the next 5 years, there was a 4-fold increase in CTPA use and a 33% decrease in V/Q scanning [6]. However, CTPA is associated with a nearly 7-fold higher radiation burden than V/Q scanning [6], with attributable lifetime malignancy risk of up to 2.76% in young female patients [7]. Moreover, in a recent prospective study, it was found that up to 14% of patients who underwent CTPA developed contrast-induced nephropathy [8].

Increased rates of CTPA use and improved understanding of the associated adverse effects have prompted researchers to measure the CTPA yield [9-11]. The CTPA yield is a measure of the appropriateness of use, defined as the percentage of tests completed to evaluate for PE that are positive for PE. The majority of these studies have used manual chart abstraction to calculate the CTPA yield [9-11]. Furthermore, a form of artificial intelligence, natural language processing, has been shown to reliably calculate the CTPA yield in a few recent studies [12-14].

These methods have demonstrated reliability but are time consuming or require technology not available at most health care institutions. To date, a simple, standardized method of electronically calculating the CTPA yield has not been described. The objective of this study is to propose and validate a computerized method for calculating the CTPA yield in the emergency department (ED).

## Methods

### Procedure

We performed a multicenter observational study to validate a computerized method of calculating CTPA yield. The study was conducted at two tertiary care hospitals, the North Shore University Hospital and the Long Island Jewish Medical Center in New York, in April and November, 2016, respectively. The two hospitals are supported by the Sunrise Clinical Manager electronic health record (EHR), a subsidiary of Allscripts Healthcare Solutions (Chicago, Illinois, United States). This study was approved by the Northwell Health’s Institutional Review Board.

**Figure 1:**
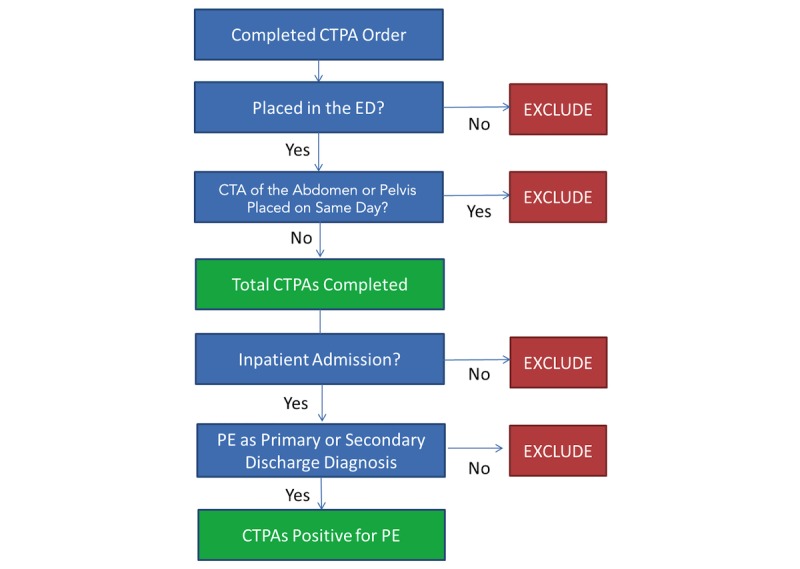
Algorithm of the computerized method for measuring computed tomography pulmonary angiography (CTPA) yield. ED: emergency department; CTA: computed tomography angiography; PE: pulmonary embolism.

The EHR databases at the two institutions are the replicated copies of the Sunrise Clinical Manager application. The database is replicated near real time with a <2-hour latency. Of note, this process is monitored by dedicated database administrators and analytics support team members to ensure fidelity. The databases were queried for CTPA orders completed in the ED over a 1-month period for each hospital. Patients’ visits were extracted from the EHR if they had a “completed” CTPA order during their ED course. However, patients with “cancelled” or “discontinued” CTPA orders were not included. Furthermore, patients with CTPAs ordered on the same day as CT angiography of the abdomen and pelvis were excluded, as these were under the protocol to rule out aortic dissection and not PE (Figure 1).

CTPA orders from the ED were then linked to inpatient visits. PE diagnosis was measured on the basis of an inpatient discharge diagnosis of the International Classification of Diseases, Clinical Modification codes, versions 9 and 10 (ICD-9-CM and ICD-10-CM), provided by the Centers for Medicare and Medicaid Services and the National Center for Health Statistics. We included both primary and secondary diagnoses in the analysis. Furthermore, the full range of PE diagnosis codes was used: 415.0, 415.11, 415.12, 415.13, and 415.19 for ICD-9-CM; and I26.0, I26.01, I26.02, I26.09, I26.9, I26.90, I26.92, and I26.99 for ICD-10-CM.

The CTPA yield was calculated as the number of ED CTPA orders linked to an inpatient discharge diagnosis of PE divided by the total number of CTPAs completed in the ED that month. This calculated yield was then validated by performing a manual chart review. In the manual chart review, the free-text radiology read of each completed ED CTPA order was reviewed to classify the CTPA as positive or negative for PE. In addition, both the ED provider note and inpatient discharge note were reviewed to ensure that the CTPA was done to evaluate for PE and the diagnosis was not revised during the inpatient visit.

The computerized calculated yield for each month and institution was compared with the yield generated from manual chart reviews by 2 independent reviewers. The reviewers were trained internal medicine physicians with experience in reading radiology reports. Of note, the reviewers were blinded to each other but not to the computerized results and had full access to discharge documentation and the entire medical chart.

### Data Analysis

We used McNemar’s test to determine whether the CTPA yields were different between the computerized calculated yield and the manual chart reviews. The kappa coefficient and the corresponding 95% CI were calculated to measure the agreement between the computerized calculated yield and the manual chart reviews.

## Results

In total, 375 CTPAs were completed during the allotted period for review. Of them, 6.9% (26/375) orders were completed on the same day as a CT angiography of the abdomen and pelvis and were, thus, excluded. Furthermore, manual chart reviews confirmed that each of these 26 omitted CTPA orders and none of the remaining 349 were ordered with the intention to rule out an aortic dissection. There were no cases where a chart review of the ED provider note and inpatient discharge note changed the interpretation of the CTPA results. In addition, the independent reviewers reported the same results, with no disagreement.

At the North Shore University Hospital site, of 203 CTPA orders completed for the evaluation of PE, 18 orders were found to have an associated inpatient discharge diagnosis of PE. The calculated yield was 8.9% (18/203). Manual chart reviews revealed 19 positive scans for a true CTPA yield of 9.4% (19/203). Notably, one discordant scan was found in a patient directly discharged from the ED, and as a result, the patient never received an inpatient discharge diagnosis.

At the Long Island Jewish Medical Center site, 146 CTPA orders were completed and 9 were found to have an associated inpatient discharge diagnosis of PE. The calculated CTPA yield was 6.2% (9/146). The manual chart reviews produced identical results, confirming 9 positive CTPA scans.

**Figure 2:**
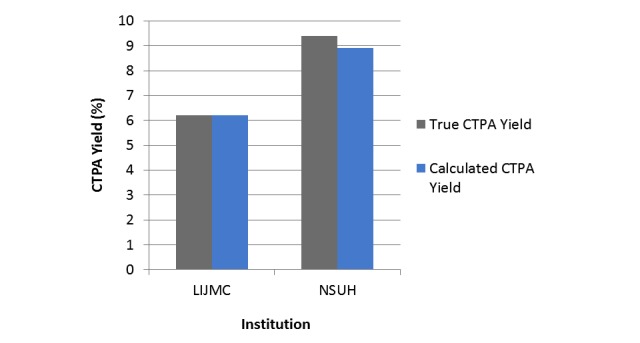
True computed tomography pulmonary angiography (CTPA) yield vs calculated CTPA Yield by computerized method. LIJMC: Long Island Jewish Medical Center; NSUH: North Shore University Hospital.

Overall, the computerized method captured 27 of 28 scans positive for PE, with an accuracy of 96.4% (27/28; Figure 2). The overall CTPA yield for both institutions was 7.7% (27/349). In this study, the *P*=.32, indicating that the proportions were not significantly different between the two groups. Furthermore, the kappa coefficient was .98, with 95% CI (0.94-1.00) also indicating an agreement between the two groups.

## Discussion

### Principal Findings

To the best of our knowledge, this is the first study to propose and validate a simple, standardized method of electronically calculating the CTPA yield. This method has wide applicability to address increasing concerns about both overtesting and overdiagnosis of PE. The increase in the incidence of PE accompanying the increased use of CTPA [15] has been associated with a decrease in the PE case mortality [16,17]. Physicians are testing more for PE and seem to be finding and treating clinically insignificant PEs. The ability of health care systems to computerize the monitoring of the CTPA yield allows them to address overtesting and overdiagnosis using systems interventions.

In addition, clinical decision support tools, built to estimate the pretest probability of PE and discourage the CTPA use in low-risk patients, have been shown to improve the CTPA yield. These tools reduce testing by 25%, without any missed PEs [9,18,19]. However, these studies are limited by the time required for manual chart reviews. Studies of interventions designed to reduce unnecessary CTPA use decrease exposure to both contrast and radiation and avoid costly “incidentalomas.” Furthermore, incidental findings requiring clinical or radiological follow-up were found in 24% of patients without PE [16].

In this study, we describe a validated method to measure the CTPA yield that allows the data collection process to be computerized and does not require artificial intelligence. We utilized both ICD-9 and ICD-10 codes to fully encompass PE coding at the time of discharge. This method can be applied to allow for the comparison of the CTPA yield in different health care systems and different types of acute care facilities. Improved data collection will allow for more targeted interventions, with an ultimate goal of increased CTPA yields and decreased CTPA utilization.

### Limitations

The one discordant scan in our study points to a limitation that will likely become more relevant in future studies. One positive CTPA was missed by our computerized method because the patient was discharged directly from the ED, meaning there was no linkable inpatient visit or potential discharge ICD code. With a push toward cost-conscious care and away from inpatient medicine, there will likely be more patients with acute PE diagnosed in the ED who are treated as outpatients. While the safety of this practice was unclear and controversial just a few years ago [20,21], it has recently become more common with the increased use of direct-acting oral anticoagulants [22,23] and safety research in the field [24,25]. This is particularly true in large health care systems with tertiary EDs that can safely assess patients’ risk with bedside echocardiography and lower-extremity ultrasound [26]. Future studies will link CTPA scans to both inpatient and ED visits to improve the accuracy.

In addition, future studies may specify the type of PE and consider the discovery of a subsegmental PE as a negative study. This was not addressed in this study as currently, these are not treated differently and ICD codes do not distinguish these types of PEs. Notably, although this was not observed in our study, this method will likely count studies conducted in patients with chronic PE as positive. Finally, this study was conducted at two hospitals under one health care system, Northwell Health. Hence, future directions include studying this method at other institutions to ensure its accuracy.

### Conclusions

This is the first successful validation study of a simple computerized method for calculating the CTPA yield in the ED. This method for data extraction allows for an accurate and efficient determination of the CTPA yield and represents a significant improvement from the manual chart review. With this ability, health care systems can monitor the appropriate use of CTPA and the effect of interventions to reduce overuse and decrease preventable iatrogenic harm.

## Abbreviations

CTPA: Computed Tomography Pulmonary Angiography
ED: emergency department
EHR: electronic health record
ICD-9-CM: International Classification of Diseases, Clinical Modification codes, version 9
ICD-10-CM: International Classification of Diseases, Clinical Modification codes, version 10
PE: pulmonary embolism
V/Q: ventilation/perfusion lung

## References

[1] Godwin JD, Webb WR, Gamsu G, Ovenfors CO (1980). Computed tomography of pulmonary embolism. AJR Am J Roentgenol.

[2] Wiener RS, Schwartz LM, Woloshin S (2011). Time trends in pulmonary embolism in the United States: evidence of overdiagnosis. Arch Intern Med.

[3] Stein PD, Kayali F, Olson RE (2004). Trends in the use of diagnostic imaging in patients hospitalized with acute pulmonary embolism. Am J Cardiol.

[4] Stein PD, Woodard PK, Weg JG, Wakefield TW, Tapson VF, Sostman HD, Sos TA, Quinn DA, Leeper KV, Hull RD, Hales CA, Gottschalk A, Goodman LR, Fowler SE, Buckley JD, PIOPED II Investigators (2007). Diagnostic pathways in acute pulmonary embolism: recommendations of the PIOPED II Investigators. Radiology.

[5] Remy-Jardin M, Pistolesi M, Goodman LR, Gefter WB, Gottschalk A, Mayo JR, Sostman HD (2007). Management of suspected acute pulmonary embolism in the era of CT angiography: a statement from the Fleischner Society. Radiology.

[6] Mettler FA, Huda W, Yoshizumi TT, Mahesh M (2008). Effective doses in radiology and diagnostic nuclear medicine: a catalog. Radiology.

[7] Niemann T, Zbinden I, Roser HW, Bremerich J, Remy-Jardin M, Bongartz G (2013). Computed tomography for pulmonary embolism: assessment of a 1-year cohort and estimated cancer risk associated with diagnostic irradiation. Acta Radiol.

[8] Mitchell AM, Jones AE, Tumlin JA, Kline JA (2012). Prospective study of the incidence of contrast-induced nephropathy among patients evaluated for pulmonary embolism by contrast-enhanced computed tomography. Acad Emerg Med.

[9] Wang RC, Bent S, Weber E, Neilson J, Smith-Bindman R, Fahimi J (2016). The Impact of Clinical Decision Rules on Computed Tomography Use and Yield for Pulmonary Embolism: A Systematic Review and Meta-analysis. Ann Emerg Med.

[10] van Belle A, Büller HR, Huisman MV, Huisman PM, Kaasjager K, Kamphuisen PW, Kramer MHH, Kruip MJHA, Kwakkel-van EJM, Leebeek FWG, Nijkeuter M, Prins MH, Sohne M, Tick LW, Christopher Study Investigators (2006). Effectiveness of managing suspected pulmonary embolism using an algorithm combining clinical probability, D-dimer testing, and computed tomography. JAMA.

[11] Dunne RM, Ip IK, Abbett S, Gershanik EF, Raja AS, Hunsaker A, Khorasani R (2015). Effect of Evidence-based Clinical Decision Support on the Use and Yield of CT Pulmonary Angiographic Imaging in Hospitalized Patients. Radiology.

[12] Swartz J, Koziatek C, Theobald J, Smith S, Iturrate E (2017). Creation of a simple natural language processing tool to support an imaging utilization quality dashboard. Int J Med Inform.

[13] Tian Z, Sun S, Eguale T, Rochefort C (2017). Automated Extraction of VTE Events From Narrative Radiology Reports in Electronic Health Records: A Validation Study. Med Care.

[14] Rochefort CM, Verma AD, Eguale T, Lee TC, Buckeridge DL (2015). A novel method of adverse event detection can accurately identify venous thromboembolisms (VTEs) from narrative electronic health record data. J Am Med Inform Assoc.

[15] Hall WB, Truitt SG, Scheunemann LP, Shah SA, Rivera MP, Parker LA, Carson SS (2009). The prevalence of clinically relevant incidental findings on chest computed tomographic angiograms ordered to diagnose pulmonary embolism. Arch Intern Med.

[16] Perrier A, Bounameaux H (2006). Accuracy or outcome in suspected pulmonary embolism. N Engl J Med.

[17] Glassroth J (2007). Imaging of pulmonary embolism: too much of a good thing?. JAMA.

[18] Yan Z, Ip IK, Raja AS, Gupta A, Kosowsky JM, Khorasani R (2017). Yield of CT Pulmonary Angiography in the Emergency Department When Providers Override Evidence-based Clinical Decision Support. Radiology.

[19] Costantino MM, Randall G, Gosselin M, Brandt M, Spinning K, Vegas CD (2008). CT angiography in the evaluation of acute pulmonary embolus. AJR Am J Roentgenol.

[20] Piran S, Le GG, Wells P, Gandara E, Righini M, Rodger M, Carrier M (2013). Outpatient treatment of symptomatic pulmonary embolism: a systematic review and meta-analysis. Thromb Res.

[21] Yoo H, Queluz T, El DR (2014). Outpatient versus inpatient treatment for acute pulmonary embolism. Cochrane Database Syst Rev.

[22] Ghazvinian R, Gottsäter A, Elf JL (2018). Efficacy and safety of outpatient treatment with direct oral anticoagulation in pulmonary embolism. J Thromb Thrombolysis.

[23] Medina A, Raskob G, Ageno W, Cohen Alexander T, Brekelmans Marjolein P A, Chen Cathy Z, Grosso Michael A, Mercuri Michele F, Segers Annelise, Verhamme Peter, Vanassche Thomas, Wells Philip S, Lin Min, Winters Shannon M, Weitz Jeffrey I, Büller Harry R (2017). Outpatient Management in Patients with Venous Thromboembolism with Edoxaban: A Post Hoc Analysis of the Hokusai-VTE Study. Thromb Haemost.

[24] Abusibah H, Abdelaziz MM, Standen P, Bhatia P, Hamad MM (2018). Ambulatory management of pulmonary embolism. Br J Hosp Med (Lond).

[25] Jiménez David, Yusen R (2018). Outpatient therapy for acute symptomatic pulmonary embolism diagnosed in the emergency department: Time to improve the evidence base. Thromb Res.

[26] Bledsoe JR, Woller SC, Stevens SM, Aston V, Patten R, Allen T, Horne BD, Dong L, Lloyd J, Snow G, Madsen T, Elliott CG (2018). Management of Low-Risk Pulmonary Embolism Patients Without Hospitalization: The Low-Risk Pulmonary Embolism Prospective Management Study. Chest.

